# BER-Ago: A simultaneous detection and inhibitor screening platform for multiple base excision repair proteins

**DOI:** 10.1016/j.jpha.2025.101507

**Published:** 2025-12-01

**Authors:** Sheng Ding, Ju Chen, Jing Li, Ting Zhu, Wenyue Jia, Shiqi Xiao, Jin Yang, Dianxiang Lu

**Affiliations:** a Clinical Medical College & Affiliated Hospital, Chengdu University, Chengdu, 610106, China; b Laboratory of Extreme Environment Medical Engineering and Applied Innovation, Chengdu University, Chengdu, 610106, China

## Abstract

• BER-Ago was proposed to detect four non-nucleic acid targets simultaneously.
• BER-Ago assay could be repurposed to evaluate and screen potential inhibitors for four BER proteins.
• BER-Ago offers a new insight into the developments of Ago-based tools for biosensing and therapeutic applications.

## Graphical abstract

**Figure:**
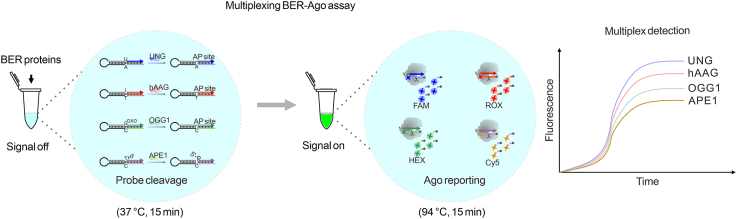

Base excision repair (BER) is a critical DNA repair pathway responsible for correcting base lesions, thereby playing an indispensable role in maintaining genomic integrity. The key BER enzymes, including uracil-DNA glycosylase (UNG), human alkyladenine DNA glycosylase (hAAG), 8-oxoguanine DNA glycosylase 1 (OGG1), and apurinic/apyrimidinic (AP) endonuclease 1 (APE1), are crucial. Their dysregulation is mechanistically linked to oncogenesis and chronic inflammation, making them highly attractive not only as diagnostic and prognostic biomarkers but also as therapeutic targets, particularly in the context of synthetic lethality for cancer treatment [1]. Despite their clear significance, the study and therapeutic targeting of these enzymes have been constrained by technological limitations. Conventional detection methods, such as enzyme-linked immunosorbent assay (ELISA), high performance liquid chromatography (HPLC), and mass spectrometry (MS), are often laborious, time-consuming, and costly, making them unsuitable for high-throughput applications [2]. Although clustered regularly interspaced short palindromic repeats (CRISPR)-based biosensors have emerged as sensitive detection tools, their utility for multiplexed protein detection is inherently limited by the expensive guide RNAs and, more critically, by the promiscuous trans-cleavage activity of CRISPR-associated (Cas) enzymes (e.g., Cas12a and Cas13a), which causes severe cross-talk between channels and impedes reliable multiplexing. In this context, prokaryotic Argonaute (pAgo) proteins present a compelling alternative. pAgo utilizes short DNA guides (much cheaper than RNA) and, most importantly, exhibits stringent sequence-specific cleavage activity without promiscuous trans-cleavage, making it an ideal candidate for developing highly multiplexed, low-cost biosensing platforms [3]. Considering these advantages of pAgo over other signal amplifiers, we chose PfAgo, a well-established pAgo, to develop new sensing tools for BER proteins. In this study, we introduce BER-Ago, a novel, rapid, and cost-effective platform that leverages the programmable nuclease PfAgo for simultaneous detection and inhibitor screening of four BER enzymes in a single reaction. Unlike enzyme-free amplification approaches, BER-Ago operates within 30 min, requires only short DNA guides, and achieves high sensitivity and specificity without the need for thermal cycling or multiple washing steps. BER-Ago effectively addresses the existing gap in multiplexed BER protein analysis, offering a streamlined workflow that opens new avenues for high-throughput research and drug discovery.

The principle of BER-Ago is illustrated in Fig. 1A, BER enzymes cleave their cognate modified base in stem-loop probes, creating AP sites. When the reaction was heated to 94 °C, the AP sites undergone rapid hydrolysis, releasing 15-nt 5′-phosphorylated guide DNAs. Then, the PfAgo-guide complex was formed and exerted cleavage activity towards complementary single-stranded DNA (ssDNA) reporters, generating multiplexed fluorescent signals. We first examined the feasibility of the method. As shown in Fig. 1B, robust fluorescence signal increases were observed after adding the BER enzymes to the reaction tubes containing their corresponding stem-loop probes (Table S1). Besides, the denatured polyacrylamide gel electrophoresis (PAGE) (Fig. S1) also confirmed reporter cleavage only when probe, enzyme, and PfAgo were present. Since the length of recognition site (guide DNA) plays a vital role in PfAgo activation, we then optimized the recognition site length of each probe to obtain the best signal output. As shown in Fig. S2, the 15-nt recognition site length in probe displayed the highest signal-to-noise ratios. To be noted, the positive signal disappeared when the length of recognition site was shorter than 15-nt, indicating that the guide DNA length should be longer than 15-nt to ensure the PfAgo activation, which was consistent with previous reports.

**Fig. 1:**
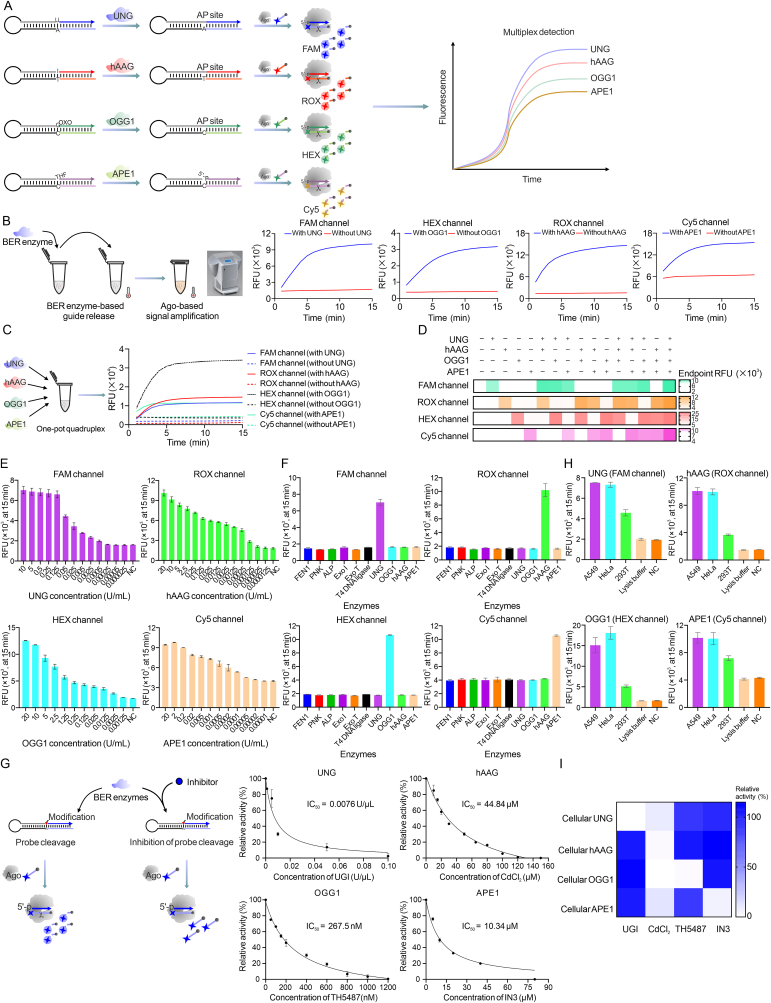
Base excision repair (BER)-argonaute (Ago) for multiplex BER protein detection and inhibitor screening. (A) Schematic illustration of the BER-Ago assay. (B) Establishment of the singleplex BER-Ago assay: the workflow of the method and detections of uracil-DNA glycosylase (UNG), 8-oxoguanine DNA glycosylase 1 (OGG1), human alkyladenine DNA glycosylase (hAAG), and apurinic/apyrimidinic (AP) endonuclease 1 (APE1) with the method. (C) Development of the quadruplex BER-Ago assay (left) and the amounts of UNG, hAAG, OGG1, and APE1 were 10, 10, 5, and 2 U/mL, respectively (right). (D) The orthogonal experiment of quadruplex BER-Ago assay by adding different BER protein combinations. (E) Analytical performance of the BER-Ago assay. The endpoint fluorescent intensity of fluorescence channels when adding serially diluted BER proteins. (F) The specificity assay of the quadruplex BER-Ago assay. The amounts of flap endonuclease 1 (FEN1), polynucleotide kinase (PNK), alkaline phosphatase (ALP), exonuclease I (Exo I), exonuclease T (Exo T), T4 DNA ligase, UNG, OGG1, hAAG, and APE1 were 16, 50, 25, 20, 250, 250, 10, 5, 10, and 2 U/mL, respectively. The error bars were obtained from three replicates. (G) Inhibition assay with the quadruplex BER-Ago system. The concentration of UNG, hAAG, OGG1, and APE1 were fixed at 0.05, 0.125, 0.25, and 0.0005 U/mL, respectively. All the error bars were obtained from three replicates. (H) Detection of cellular BER proteins with the quadruplex BER-Ago system. The endpoint fluorescent intensity of different channels when adding different cell extracts. (I) Inhibition of cellular BER proteins with different inhibitors. The concentrations of UNG inhibitor (UGI), cadmium chloride (CdCl_2_), 4-(4-bromo-2-oxo-3H-benzimidazol-1-yl)-N-(4-iodophenyl)piperidine-1-carboxamide (TH5487), and N-(3-(benzo[d]thiazol-2-yl)-6-isopropyl-4,5,6,7-tetrahydrothieno[2,3-c]pyridin-2-yl)acetamide (IN3) were 0.5 U/μL, 150 μM, 0.5 μM and 100 μM, respectively. All the error bars were obtained from three replicates. THF: tetrahydrofuran; FAM: 6-carboxyfluorescein; ROX: carboxy X-rhodamine; HEX: hexachlorofluorescein; Cy5: cyanine 5; RFU: relative fluorescence units; NC: negative control without adding BER proteins; IC_50_: the half-maximal inhibitory concentration.

Having established the singleplex BER-Ago assay, we intended to develop the multiplex detection system. We systematically optimized the buffer, probes, ssDNA reporters, Mn^2+^, and PfAgo (Supplementary data, Figs. S3–S7). Subsequently, we performed a one-pot quadruplex detection assay targeting UNG, hAAG, OGG1, and APE1. The results, depicted in Fig. 1C, demonstrated that each enzyme specifically increased fluorescence in its respective channel (6-carboxyfluorescein (FAM), carboxy X-rhodamine (ROX), hexachlorofluorescein (HEX), and cyanine 5 (Cy5)). These results confirmed the feasibility of detecting these four BER enzymes within a single reaction. To examine the potential cross-reactivity among these four proteins, we conducted an orthogonal experiment. As illustrated in the fluorescence heatmap (Fig. 1D), the introduction of each target BER protein resulted in a fluorescent signal exclusively in its corresponding channel. Furthermore, the simultaneous addition of multiple BER proteins (in duplex, triplex, and quadruplex combinations) successfully elicited multiple positive signals in the respective fluorescent channels, indicating no cross-reactivity among these BER proteins. Taken together, these results demonstrated the successful development of a multiplex detection method utilizing the BER-Ago assay.

The analytical performance of the multiplex BER-Ago assay was tested under the optimized concentrations. The dose–dependent curves displayed linear relationships between the endpoint fluorescence and the logarithm of BER enzyme concentration (Figs. 1E, S8, and S9). The limits of detection (LODs) for UNG, hAAG, OGG1, and APE1 were determined to be 9.87 × 10^−5^, 1.45 × 10^−4^, 1.68 × 10^−3^, and 3.26 × 10^−5^ U/mL, respectively (Fig. S9). The sensitivity of the multiplex BER-Ago assay was found to be comparable to many existing methods, particularly in comparison to various CRISPR-based biosensors (Table S2). Notably, our method offers enhanced multiplexing capability, while ensuring cost-effectiveness and reduced detection time. Next, the high specificity of the method was verified using non-target enzymes related to DNA manipulation (Fig. 1F).

The development of an inhibitor evaluation platform for BER proteins holds significant promise for the treatment of various disorders. To assess the feasibility of using BER-Ago for inhibitor assays, we employed several known inhibitors to target specific BER proteins. Figs. 1G and S10 demonstrated that the fluorescence intensity decreased in correlation with the reduced concentration of model inhibitors and the half-maximal inhibitory concentrations (IC_50_) were estimated, indicating a concentration-dependent inhibition of these BER proteins. Considering the simplicity and multiplexity of our BER-Ago platform, we believe this method could be an ideal alternative for the development of such inhibitors that can simultaneously target these four different BER proteins when combined with the high-throughput signal detecting platform such as microplate reader (Fig. S11).

To evaluate the feasibility of our method in biological samples, the cellular BER proteins were detected using cellular extracts. Cellular extracts produced strong multiplex signals in A549 and HeLa cells, with lower signals in 293T cells (Figs. 1H and S12), consistent with cancer-associated BER protein overexpression [4]. Besides, the results were confirmed by commercial ELISA kits (Fig. S13), demonstrating the high accuracy of our method. Furthermore, quantitative dilution (Figs. S14 and S15) and recovery experiment using cell lysate and 10% serum (Tables S3–S5) confirmed that our method could be used for quantitatively detecting cellular BER proteins with high stability and good reproducibility. Notably, the ELISA kits were unable to detect cellular BER proteins present at low abundance, whereas the BER-Ago method successfully identified these proteins, demonstrating the superior sensitivity of our approach (Fig. S14). We also conducted the inhibition assay with A549 cell lysates. The results in Figs. 1I and S16 demonstrated that the respective inhibitors effectively suppressed the corresponding BER proteins. Notably, the introduction of cadmium chloride (CdCl_2_) resulted in a decrease in fluorescence across all four fluorescent channels, highlighting its potential as a broad-spectrum BER inhibitor. These data validated the potential of our method in detecting real-life samples.

In summary, we have successfully developed BER-Ago, a multiplex biosensing platform that effectively addresses the long-standing challenge of simultaneously profiling the activities of multiple key BER enzymes. By ingeniously coupling the specific recognition of BER enzymes with the programmable cleavage activity of PfAgo, our method achieves rapid, sensitive, and highly specific detection in a one-pot format. The most significant advantage of BER-Ago over CRISPR-based methods is its inherent suitability for multiplexing, driven by the precise DNA guide-dependent cleavage of PfAgo that eliminates cross-talk, combined with substantial cost savings. We have rigorously demonstrated its robust analytical performance, its practical applicability in complex biological samples like cell extracts, and its utility as a powerful inhibitor screening platform. The ability to quantitatively assess the effect of inhibitors on multiple BER targets concurrently positions BER-Ago as a promising tool for accelerating drug discovery, especially in the development of synthetic-lethality-based cancer therapies. Importantly, the modular design principle of BER-Ago is not limited to these four enzymes; the platform can be readily extended to other glycosylases or DNA-modifying enzymes by designing corresponding probes. Beyond its immediate application in BER protein analysis, this study serves as a key example of employing Ago-based biosensors for the detection of non-nucleic acid targets, thereby expanding the toolbox for diagnostic and bioanalytical applications. Future directions could involve integrating this biochemical assay with portable detection devices, such as WaveFlex biosensors [5], to pave the way for point-of-care diagnostic applications.

## CRediT authorship contribution statement

**Sheng Ding:** Writing – review & editing, Writing – original draft, Software, Methodology, Investigation, Funding acquisition, Formal analysis, Data curation, Conceptualization. **Ju Chen:** Writing – original draft, Methodology, Investigation, Formal analysis, Data curation. **Jing Li:** Writing – original draft, Software, Methodology, Investigation, Formal analysis, Data curation. **Ting Zhu:** Software, Investigation, Data curation. **Wenyue Jia:** Investigation, Formal analysis. **Shiqi Xiao:** Investigation, Data curation. **Jin Yang:** Writing – original draft, Supervision, Resources, Project administration, Investigation, Funding acquisition. **Dianxiang Lu:** Writing – original draft, Supervision, Resources, Project administration, Methodology, Conceptualization.

## Declaration of competing interest

The authors declare that they have no known competing financial interests or personal relationships that could have appeared to influence the work reported in this paper.
