# Supplementary material for: BER-Ago: A simultaneous detection and inhibitor screening platform for multiple base excision repair proteins
Source: J Pharm Anal. 2025 Dec 1;16(5):101507. doi: 10.1016/j.jpha.2025.101507 (PMC13199826; doi:10.1016/j.jpha.2025.101507)
Supplement: Multimedia component 1 [file mmc1.docx]

***Supporting information***

**BER-Ago: A simultaneous detection and inhibitor screening platform for multiple base excision repair proteins**

**Experimental Section**

**Materials.**

The PfAgo and PfAgo reaction buffer were purchased from Jiaohong Biotech (Shanghai, China). Uracil-N-glycosylase (UNG), Alkaline Phosphatase (ALP) and T4 DNA ligase were obtained from Beyotime Biotechnology (Shanghai, China). T4 Polynucleotide Kinase (PNK) was purchased from Thermo Fisher Scientific (USA). Human alkyl adenine glycosylase (hAAG), thermostable 8-Oxoguanine DNA glycosylase 1 (OGG1), apurinic/apyrimidinic endonuclease 1 (APE1), flap endonuclease 1 (FEN1), DNA exonuclease I (Exo I), DNA exonuclease T (Exo T), Uracil Glycosylase Inhibitor (UGI), rCutSmart (B6004S), NEB buffer 4 (B7004S), UNG buffer (B0280S) and ThermoPol® Reaction buffer (B9004S) were obtained from New England Biolabs (Shanghai, China). CdCl_2_, OGG1 inhibitor TH5487 and APE1 inhibitor IN3 were obtained from Tang’s Lab. Cell lines (A549, HEK293T, and HeLa cells) were purchased from the cell bank of the Committee on Type Culture Collection of the Chinese Academy of Sciences (CTCC, Shanghai, China).

**Stem-loop probe and reporter treatment.**

The oligonucleotide sequences were synthesized and purified by Sangon Biotech (Shanghai, China). The stem-loop DNA probes and DNA reporters were measured using a NanoDrop UV-Vis spectrophotometer (Thermo Fisher Scientific, USA) and diluted with sterile water. Before use as input, all stem-loop probes were heated at 90 °C for 5 minutes and cooled down to room temperature. Then, the probes and reporters were stored at -20 °C until use. All the oligonucleotide sequences used in this study were presented in **Table S1.**

**Singleplex detection for individual protein.**

For UNG test, 10 μL of the UNG-based probe cleavage reaction contained 1 × UNG buffer (20 mM Tris-HCl, 1 mM DTT, 1 mM EDTA (pH 8 at 25 °C)), 500 nM UNG stem-loop probe, and 10 U/mL UNG. For hAAG test, 10 μL of the hAAG-based probe cleavage reaction contained 1 × ThermoPol® Reaction buffer (20 mM Tris-HCl, 10 mM (NH_4_)_2_SO_4_, 10 mM KCl, 2 mM MgSO_4_, 0.1% Triton® X-100 (pH 8.8 at 25 °C)), 500 nM hAAG stem-loop probe, and 10 U/mL hAAG. For OGG1 test, 10 μL of the OGG1-based probe cleavage reaction contained 1 × rCutSmart buffer (50 mM potassium acetate, 20 mM tris-acetate, 10 mM magnesium acetate, 100 µg/mL recombinant albumin, (pH 7.9 at 25 °C)), 500 nM OGG1 stem-loop probe, and 5 U/mL OGG1. For APE1 test, 10 μL of the APE1-based probe cleavage reaction contained 1 × NEB buffer 4 (50 mM potassium acetate, 20 mM tris-acetate, 10 mM magnesium acetate, 1 mM DTT, (pH 7.9 at 25 °C)), 500 nM APE1 stem-loop probe, and 2 U/mL APE1. The cleavage reactions were carried out at 37 °C for 15 min. Then the PfAgo-based signal reporting reaction was added to the above BER protein-based cleavage reaction. Finally, a total of 20 uL PfAgo-based signal reporting reactions comprised 0.5 uL PfAgo, 2 mM of MnCl2, 1 × PfAgo reaction buffer, 500 nM of specific reporter DNA (FAM-labeled reporter for UNG test, HEX-labeled reporter for OGG1 test, ROX-labeled reporter for hAAG test, or Cy5-labeled reporter for APE1 test) and 10 uL of BER protein-based probe cleavage product. The signal reporting reaction was conducted at 94 ℃ for 15 min. The fluorescence signal was recorded at 1 min interval using PikoReal 24 RealTime PCR System (Thermo Fisher Scientific, USA). The excitation and emission wavelengths employed in the experiment were as follows: FAM (excitation at 475 nm and emission at 520 nm), HEX (excitation at 530 nm and emission at 560 nm), ROX (excitation at 580 nm and emission at 610 nm), and Cy5 (excitation at 630 nm and emission at 670 nm).

**Quadruplex detection.**

The quadruplex detection also contained two steps: BER protein-based probe cleavage and PfAgo-based signal reporting. The BER protein-based probe cleavage reaction (10 uL) comprised 1 × ThermoPol® Reaction buffer, 175 nM of each stem-loop probe, certain amounts of UNG, hAAG, OGG1 and APE1. After incubation at 37 ℃ for 15 min, the PfAgo-based signal reporting reaction (10 uL) was added. In a total 20 uL of reporting reaction, 0.5 uL PfAgo, 2 mM of MnCl_2_, 1 × PfAgo reaction buffer, 375 nM of FAM-labeled reporter, 250 nM of HEX-labeled reporter, 375nM of ROX-labeled reporter and 500 nM of Cy5-labeled reporter were contained. The signal reporting reaction was conducted at 94 ℃ for 15 min. The fluorescence signal was recorded at 1 min interval using PikoReal 24 RealTime PCR System.

**Sensitivity and specificity assay.**

For the sensitivity assay, UNG, hAAG, OGG1, and APE1 were serially diluted using their respective reaction buffers and subsequently added to the tetraplex BER-Ago detection reaction. The linear relationships between the endpoint fluorescence intensity and the logarithm of the BER protein concentrations were then determined. For specificity assay, 16 U/mL FEN1, 50 U/mL PNK, 25 U/mL ALP, 20 U/mL Exo I, 250 U/mL Exo T, 250 U/mL T4 DNA ligase, 10 U/mL UNG, 10 U/mL hAAG, 5 U/mL OGG1 and 2 U/mL APE1 were taken as input to the quadruplex BER-Ago detection system.

**BER proteins detection in cell extracts.**

Protein extraction was performed on A549, 293T, and HeLa cells, which served as the input for the study. Approximately 10^6 cultured cells were harvested and lysed using RIPA Lysis buffer solution (NCM-Biotech, China) on ice for 30 mins to obtain cell extracts. The resulting supernatants were centrifuged at 12,000 rpm for 10 mins and subsequently stored at -20 °C until use.

**Inhibition assay.**

In the inhibition assay, various inhibitors were employed, including UGI, CdCl_2_, TH5487 (4-(4-Bromo-2-oxo-3H-benzimidazol-1-yl)-N-(4-iodophenyl)piperidine-1-carboxamide), and IN3 (N-(3-(benzo[d]thiazol-2-yl)-6-isopropyl-4,5,6,7-tetrahydrothieno[2,3-c]pyridin-2-yl)acetamide), to target and inhibit UNG, hAAG, OGG1, and APE1, respectively. The relative activity was calculated using the formula: (Fi - Fnc) / (Fpc - Fnc), where Fi represents the endpoint fluorescence intensity of the reaction with the inhibitor, Fpc denotes the endpoint fluorescence intensity of the reaction without adding inhibitor, and Fnc indicates the endpoint fluorescence intensity of the reaction without adding BER protein. The inhibition assay for the cell extract was performed under identical conditions, with the exception that 1 µL of the diluted supernatants from A549 cell extracts was utilized as the input.

**Table S1. DNA sequences used in the study**

| Name | Sequence (from 5’ to 3’) |
| --- | --- |
| 13nt UNG probe | GCCGGGCAACACGAGAGGGCGGCGGTTTTTCCGCCGCCCTC**dU**CGTGTTGCCCGGC ^1^ |
| 15nt UNG probe | CCGCCGGGCAACACGAGAGGGCGGCGGTTTTTCCGCCGCCCTC**dU**CGTGTTGCCCGGCGG |
| 17nt UNG probe | CCGCCGGGCAACACGACAGAGGGCGGCGGTTTTTCCGCCGCCCTC**dU**GTCGTGTTGCCCGGCGG |
| 19nt UNG probe | CCGCCGGGCAACACGACGCAGAGGGCGGCGGTTTTTCCGCCGCCCTC**dU**GCGTCGTGTTGCCCGGCGG |
| 13nt hAAG probe | TTGCGGCGGTAACGAGGGCGGCGGTTTTTCCGCCGCCCTC**dI**GTTACCGCCGCAA^2^ |
| 15nt hAAG probe | GCTTGCGGCGGTAACGAGGGCGGCGGTTTTTCCGCCGCCCTC**dI**GTTACCGCCGCAAGC |
| 17nt hAAG probe | TTGCTTGCGGCGGTAACGAGGGCGGCGGTTTTTCCGCCGCCCTC**dI**GTTACCGCCGCAAGCAA |
| 19nt hAAG probe | CATTGCTTGCGGCGGTAACGAGGGCGGCGGTTTTTCCGCCGCCCTC**dI**GTTACCGCCGCAAGCAATG |
|  |  |
| 13nt OGG1 probe | CAGTGCCTCTGCGCGAGGGCGGCGGTTTTTCCGCCGCCCTC**oxoG**CGCAGAGGCACTG^3^ |
| 15nt OGG1 probe | CTCAGTGCCTCTGCGCGAGGGCGGCGGTTTTTCCGCCGCCCTC**oxoG**CGCAGAGGCACTGAG |
| 17nt OGG1 probe | AACTCAGTGCCTCTGCGCGAGGGCGGCGGTTTTTCCGCCGCCCTC**oxoG**CGCAGAGGCACTGAGTT |
| 19nt OGG1 probe | CTCAGTGCCTCTGCGGACGAGGGCGGCGGTTTTTCCGCCGCCCTC**oxoG**TCCGCAGAGGCACTGAG |
| 13 nt APE1 probe | ACCATCAATCGCTGAGGGCGGCGGTTTTTCCGCCGCCCTC**THF**AGCGATTGATGGT^4^ |
| 15 nt APE1 probe | TCACCATCAATCGCTGAGGGCGGCGGTTTTTCCGCCGCCCTC**THF**AGCGATTGATGGTGA |
| 17 nt APE1 probe | TATCACCATCAATCGCTGAGGGCGGCGGTTTTTCCGCCGCCCTC**THF**AGCGATTGATGGTGATA |
| 19 nt APE1 probe | CGTATCACCATCAATCGCTGAGGGCGGCGGTTTTTCCGCCGCCCTC**THF**AGCGATTGATGGTGATACG |
| ssDNA reporter for UNG | FAM-CACCGCCGGGCAACACGACG-BHQ1 |
| ssDNA reporter for hAAG | ROX-ACATTGCTTGCGGCGGTAAC-BHQ2 |
| ssDNA reporter for OGG1 | HEX-AACTCAGTGCCTCTGCGGAG-BHQ1 |
| ssDNA reporter for APE1 | Cy5-CCGTATCACCATCAATCGCT-BHQ2 |

^1^ The red font denoted the uracil modification;

^2^ The red font denoted the hypoxanthine modification.

^3^ The red font denoted the 8-oxoguanine modification.

^4^ The red font denoted the tetrahydrofuran modification.

**Table S2. Comparison of the analytical performance of some reported methods for BER proteins detection**

| Method strategy | Signal | Sensitivity | specificity | Operation complexity | Time | Assay temperature | Ref |
| --- | --- | --- | --- | --- | --- | --- | --- |
| Multiple Cyclic Enzymatic Repairing Amplification | fluorescence | 2.97 × 10^-4^ U/mL of OGG1 | high | moderate | 40 min | 37 ℃ | ^1^ |
| Stem-loop primer-mediated exponential amplification | fluorescence | 6.8 × 10^-4^ U/mL of UNG | high | complex | ＞2 h | 37 ℃，65 ℃ | ^2^ |
| multifunctional dsDNA probes mediated exponential rolling circle amplification | fluorescence | 1 × 10^-5^ U/mL of UNG and 2 × 10^-4^ U/mL of hAAG | moderate | simple | 40 min | 37 ℃ | ^3^ |
| EdU-CRISPR/Cas12a | Fluorescence and lateral flow strip | 2.52 × 10^-4^ U/mL of APE1, 1.48×10^−4^ U/mL of T4 PNK | high | moderate | 1 h | 37 ℃ | ^4^ |
| Strand displacement amplification (SDA) with a CRISPR/Cas12a effector | fluorescence | 4.24×10^−6^ U/mL of OGG1 | high | complex | 40 min | 37 ℃ | ^5^ |
| REST | fluorescence | 1.05×10^-5^ U/mL of APE1 | high | simple | 40 min | 37 ℃ | ^6^ |
| enzyme-free electrochemical biosensor | differential pulse voltammetry (DPV) | 6.6 × 10^−4^ U/mL of UNG | high | moderate | ＞2 h | 37 ℃ | ^7^ |
| ELISA | Absorbance at 450 nm |  |  |  |  |  |  |
| **BER-Ago** | **fluorescence** | **9.87 × 10^-5^ U/mL for UNG, 1.45 × 10^-4^ U/mL for hAAG, 1.68 × 10^-3^ U/mL for OGG1, and 3.26 × 10^-5^ U/mL for APE1** | **high** | **simple** | **30 min** | **37 ℃**  94 ℃ | **This work** |

References were listed in page S16.

**Table S3**. Detection of BER proteins spiked in cellular lysates.

| BER proteins | Content (U/mL) | Spiked (U/mL) | Detected (U/mL) | Recovery rate (%) | RSD (%) (n=3) |
| --- | --- | --- | --- | --- | --- |
| UNG | 0.0453 | 0.020 | 0.0651 | 99.0 | 4.6 |
|  | 0.0481 | 0.050 | 0.1023 | 108.4 | 3.9 |
|  | 0.0492 | 0.100 | 0.1471 | 97.9 | 4.7 |
| hAAG | 0. 1130 | 0.020 | 0.1343 | 106.5 | 4.3 |
|  | 0.1162 | 0.050 | 0.1651 | 97.8 | 5.0 |
|  | 0.1181 | 0.100 | 0.2153 | 97.2 | 4.8 |
| OGG1 | 0.5832 | 0.020 | 0.6047 | 107.5 | 4.4 |
|  | 0.6021 | 0.050 | 0.6491 | 94.0 | 4.8 |
|  | 0.5918 | 0.100 | 0.6999 | 108.1 | 5.1 |
| APE1 | 0.0172 | 0.020 | 0.03717 | 99.9 | 3.1 |
|  | 0.0201 | 0.050 | 0.0698 | 99.4 | 4.6 |
|  | 0.0196 | 0.100 | 0.1201 | 100.5 | 3.9 |

Rate of recovery = (Detected-Content)/spiked × 100 %.

**Table S4. Detection of BER proteins spiked in cellular lysates after short-term exposure (2h) at room temperature**

| BER proteins | Content (U/mL) | Spiked (U/mL) | Detected (U/mL) | Average Recovery rate (%) | RSD (%) (n=3) |
| --- | --- | --- | --- | --- | --- |
| UNG | 0.0473 | 0.020 | 0.0655 | 97.3 | 4.8 |
| hAAG | 0. 1177 | 0.020 | 0.1363 | 99.0 | 4.5 |
| OGG1 | 0.6102 | 0.020 | 0.6307 | 100.1 | 4.9 |
| APE1 | 0.0188 | 0.020 | 0.0388 | 100 | 4.1 |

Rate of recovery = (Detected-Content)/spiked × 100 %.

**Table S5**. Detection of BER proteins spiked in 10% serum.

| BER proteins | Spiked (U/mL) | Detected (U/mL) | Recovery rate (%) | RSD (%) (n=3) |
| --- | --- | --- | --- | --- |
| UNG | 0.020 | 0.0201 | 100.5 | 2.6 |
|  | 0.050 | 0.0503 | 100.6 | 1.9 |
|  | 0.100 | 0.0998 | 99.8 | 3.7 |
| hAAG | 0.020 | 0.0203 | 101.5 | 4.3 |
|  | 0.050 | 0.0511 | 102.2 | 3.0 |
|  | 0.100 | 0.1003 | 100.3 | 2.8 |
| OGG1 | 0.020 | 0.0197 | 98.5 | 2.4 |
|  | 0.050 | 0.0491 | 98.2 | 3.8 |
|  | 0.100 | 0.0999 | 99.9 | 4.1 |
| APE1 | 0.020 | 0.0191 | 98.5 | 3.9 |
|  | 0.050 | 0.0498 | 99.6 | 4.6 |
|  | 0.100 | 0.1011 | 101.1 | 3.0 |


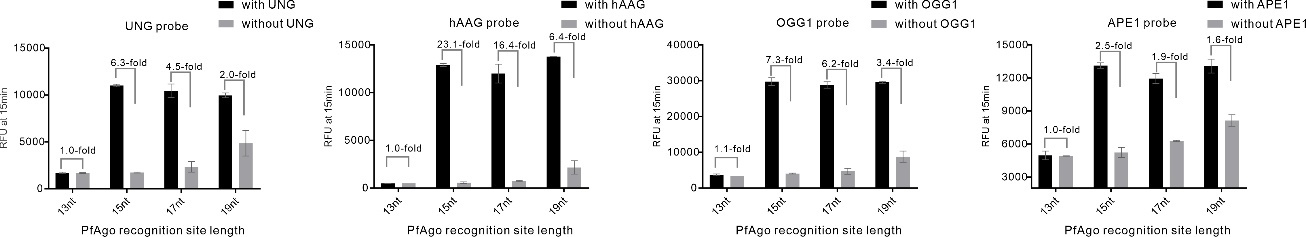


**Figure S1.** Optimization of the length of PfAgo recognition site in different probe.


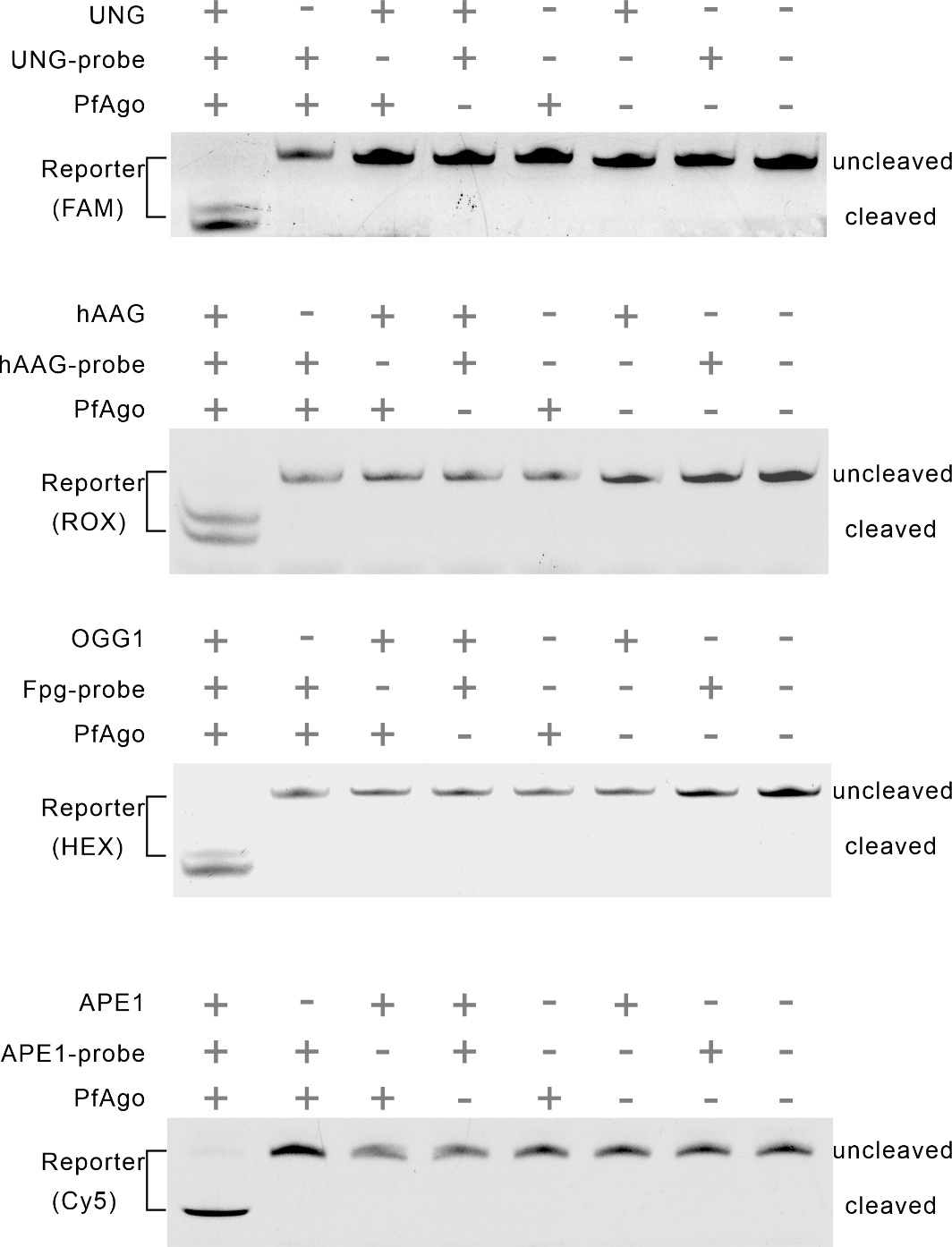


**Figure S2.** Denatured PAGE assay of the product of BER-Ago under the conditions with or without core reaction components.


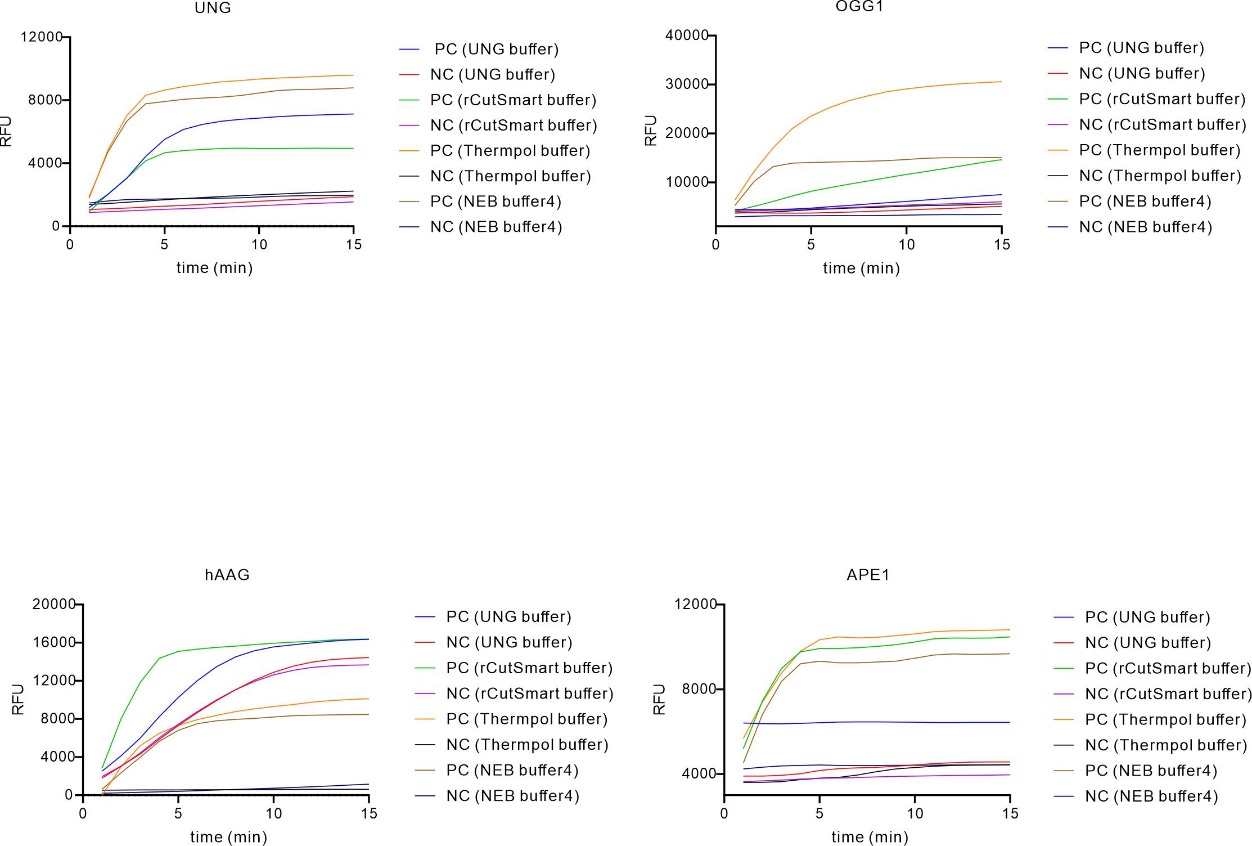


**Figure S3.** Buffer optimization for multiplex BER-Ago assay. All the experiments were conducted following the operation described in EXPERIMENTAL SECTION


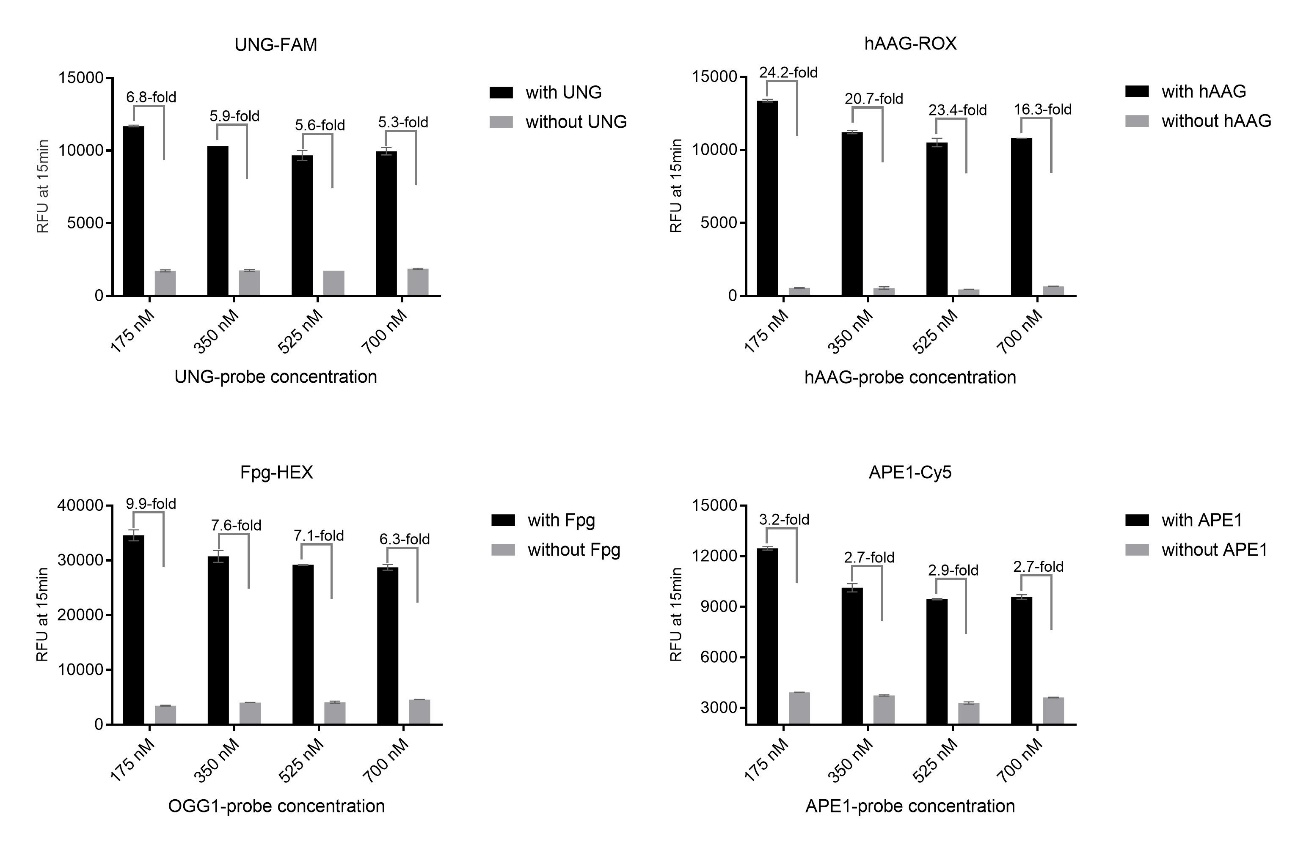


**Figure S4.** Optimization of stem-loop probe for multiplex BER-Ago assay. All the experiments were conducted following the operation described in EXPERIMENTAL SECTION


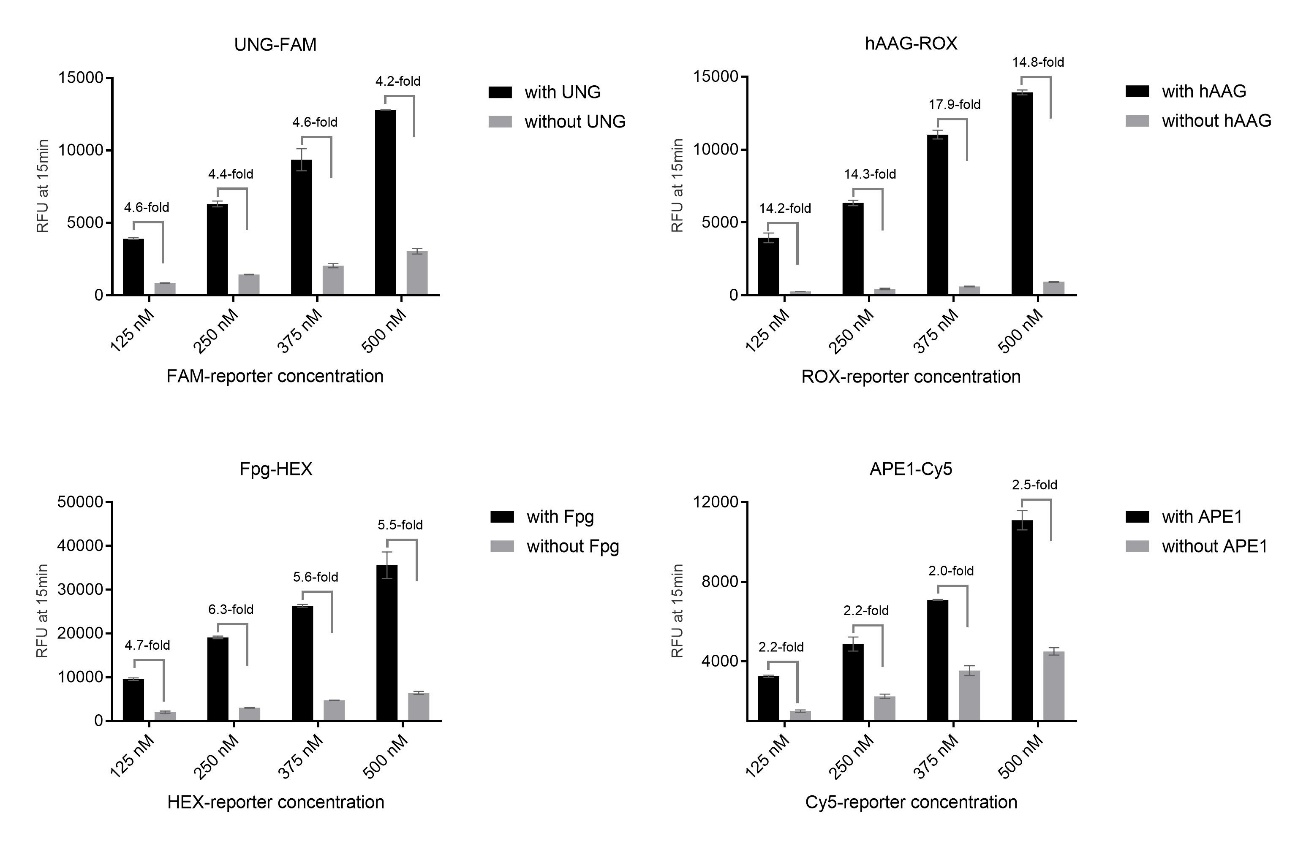


**Figure S5.** Optimization of ssDNA reporter for multiplex BER-Ago assay. All the experiments were conducted following the operation described in EXPERIMENTAL SECTION


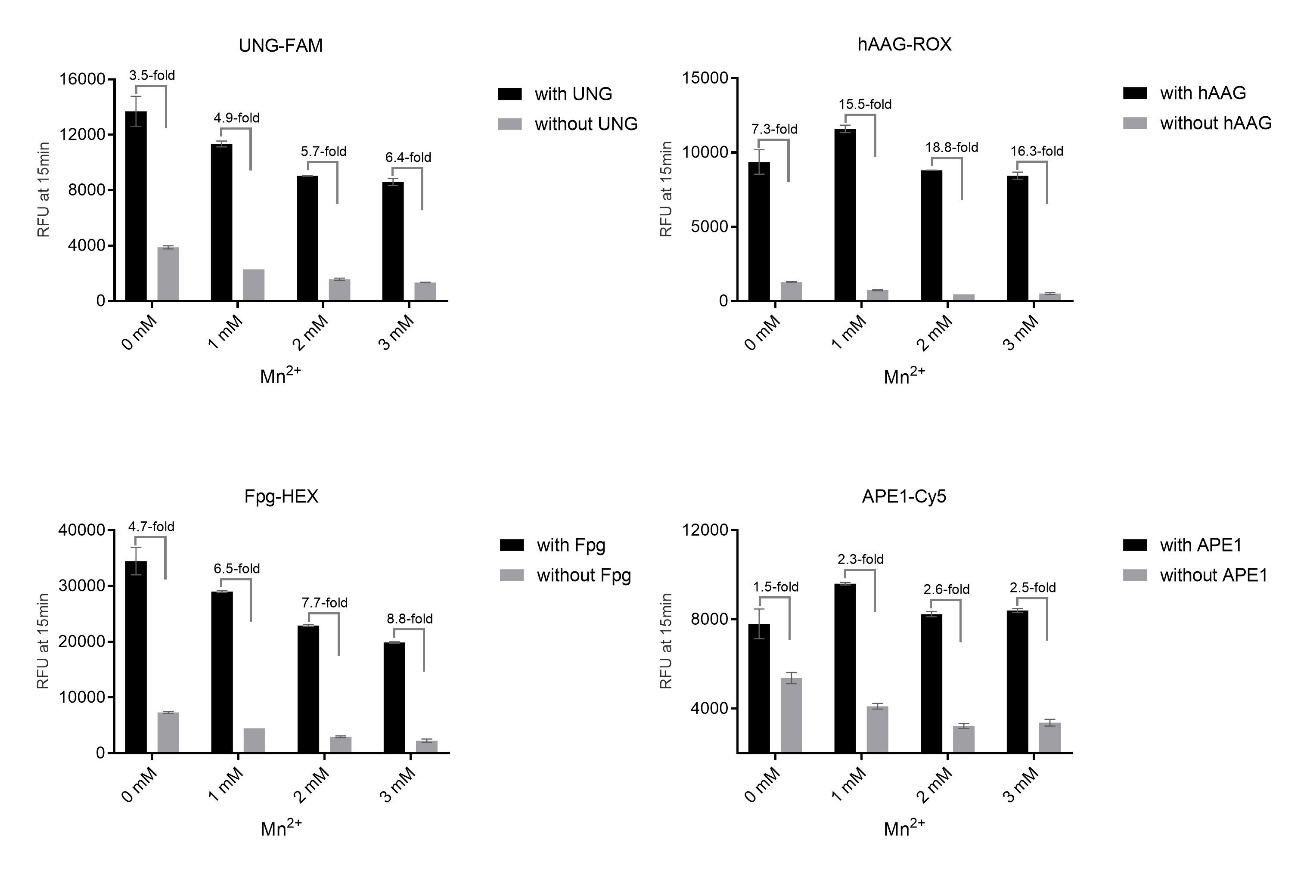


**Figure S6.** Optimization of Mn^2+^ for multiplex BER-Ago assay. All the experiments were conducted following the operation described in EXPERIMENTAL SECTION


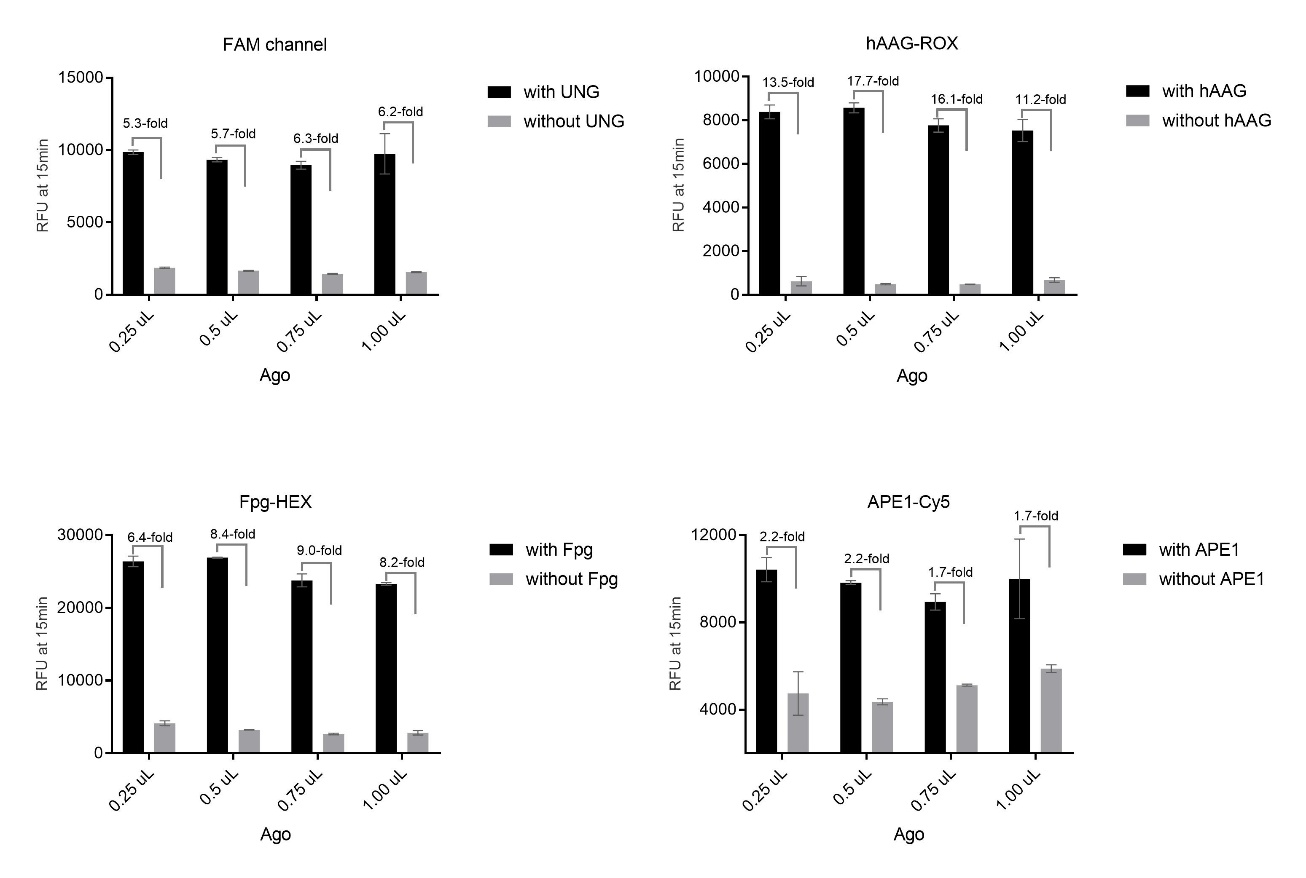


**Figure S7.** Optimization of PfAgo for multiplex BER-Ago assay. All the experiments were conducted following the operation described in EXPERIMENTAL SECTION


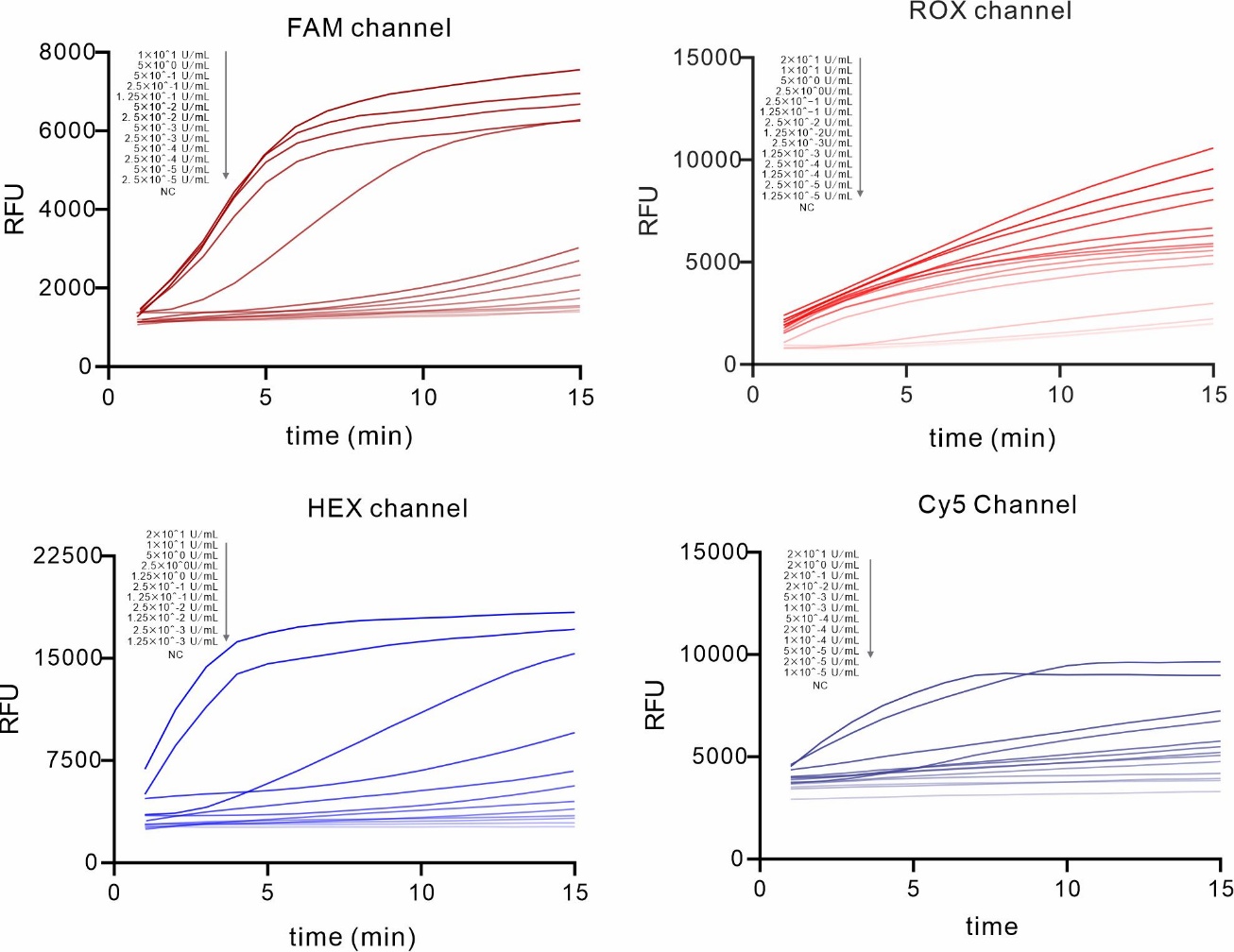


**Figure S8.** The real-time fluorescence of multiplex BER-Ago assay adding diluted UNG, hAAG, OGG1 and APE1.


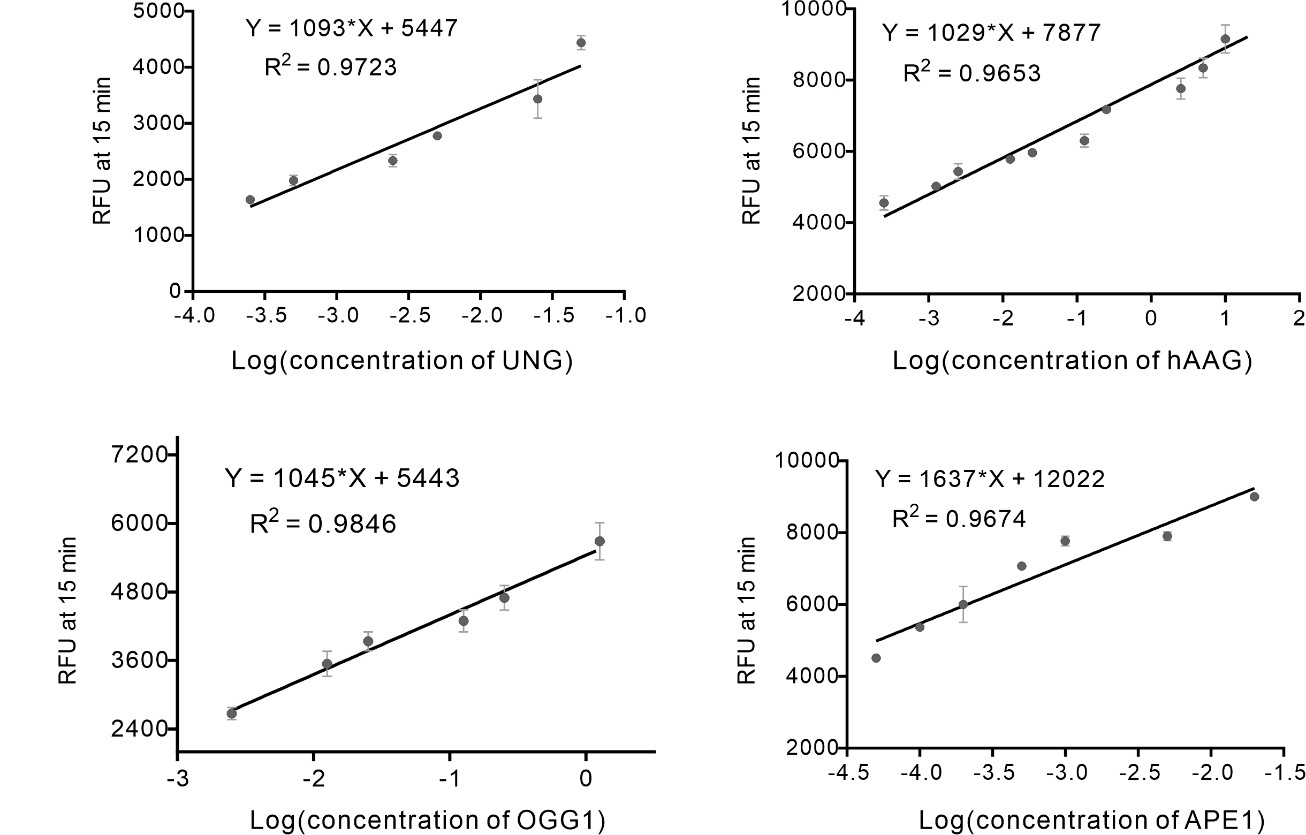


**Figure S9.** The linear relationship between the endpoint fluorescent intensity and the logarithm of BER proteins’ concentration.


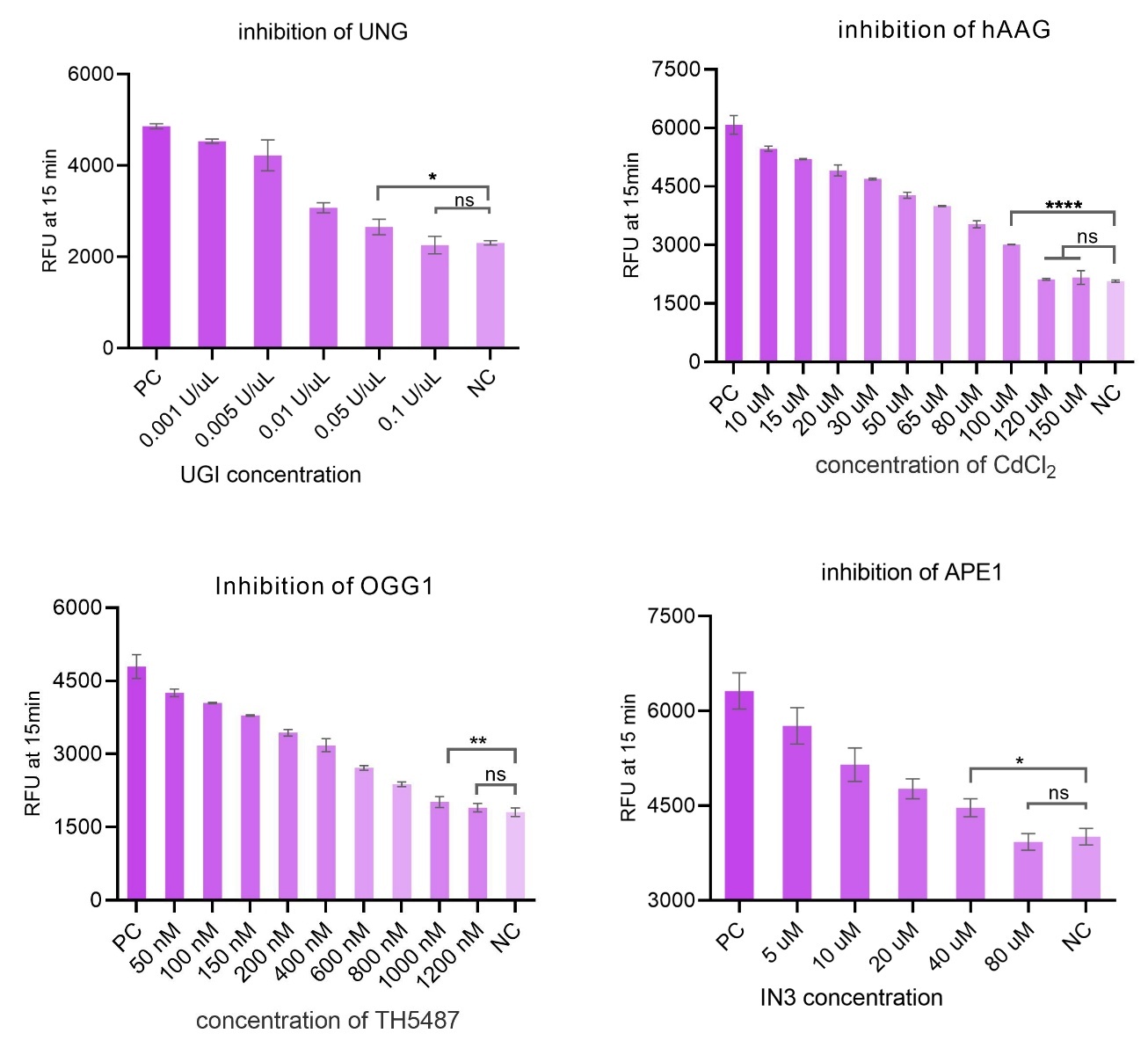


**Figure S10.** The endpoint fluorescent intensity of FAM channel when adding different amounts of UGI. The concentration of UNG was fixed at 0.05 U/mL. The endpoint fluorescent intensity of ROX channel when adding different amounts of CdCl2. The concentration of hAAG was fixed at 0.125 U/mL. The endpoint fluorescent intensity of HEX channel when adding different amounts of TH5487. The concentration of OGG1 was fixed at 0.25 U/mL. The endpoint fluorescent intensity of Cy5 channel when adding different amounts of IN3. The concentration of APE1 was fixed at 0.0005 U/mL. All the error bars were obtained from three replicates.


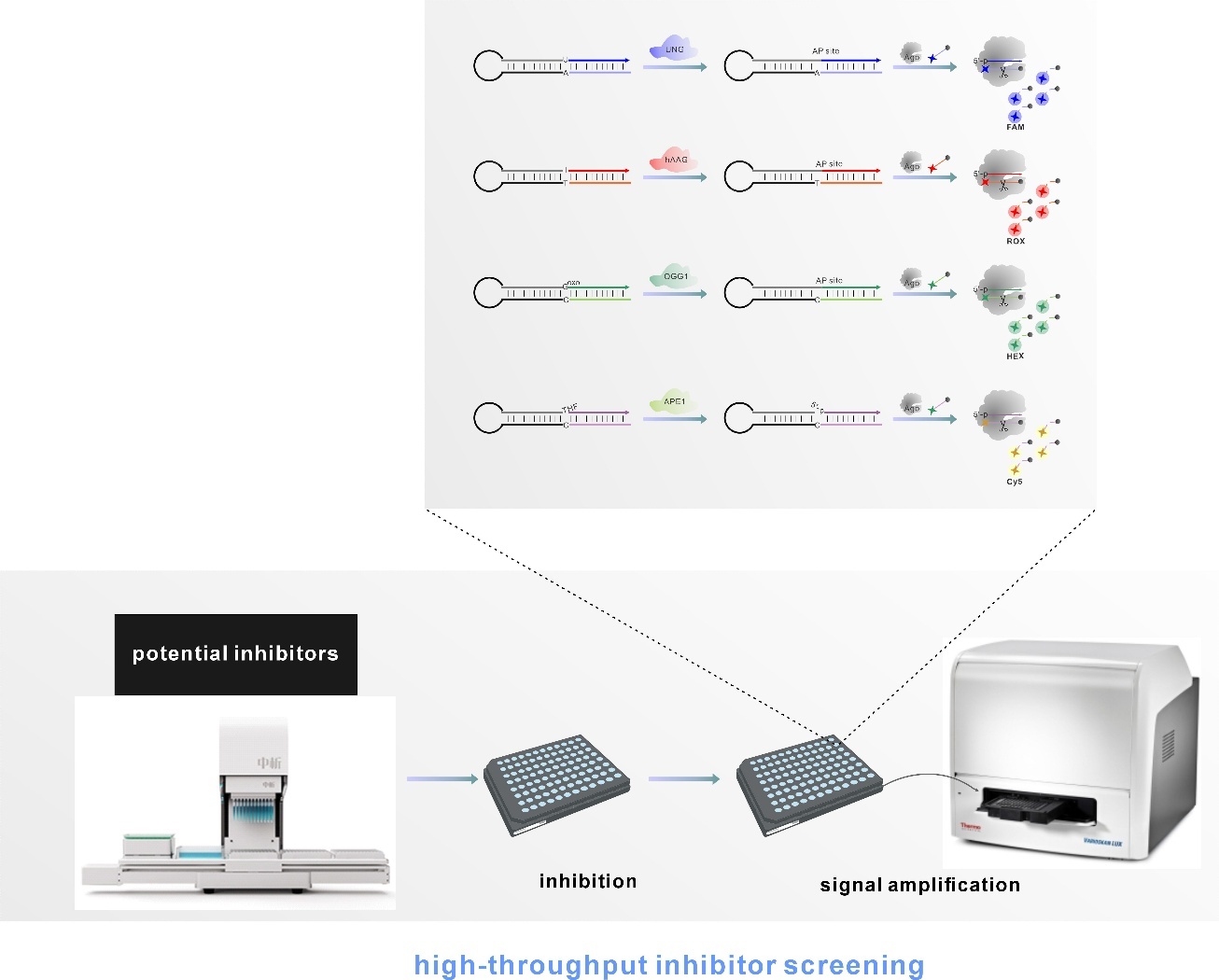


**Figure S11.** schematic illustration of high-throughput screening of inhibitors for four BER proteins.


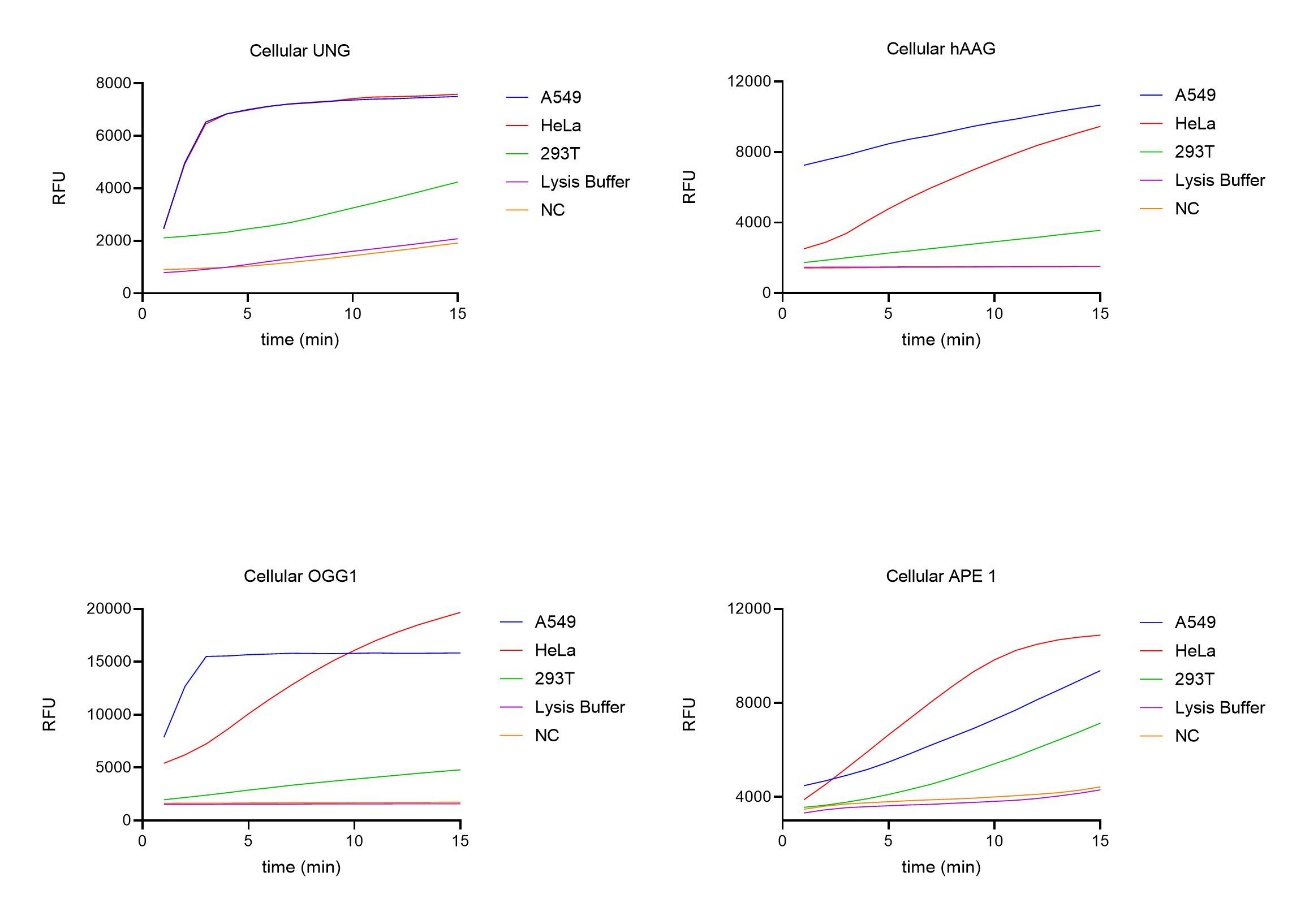


**Figure S12.** The real-time fluorescence of multiplex BER-Ago assay adding different cellular extracts.


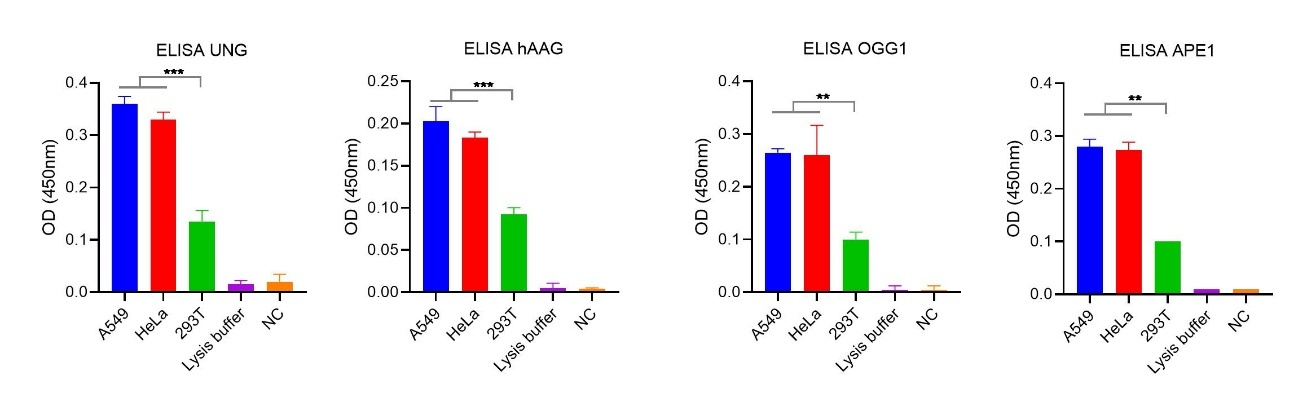


**Figure S13.** The ELISA assay of adding different cellular extracts. (The cell extracts were obtained from about 10^5^ cell counts. The total amount of protein from different cell line was fixed at 0.5 mg/mL by BCA test).


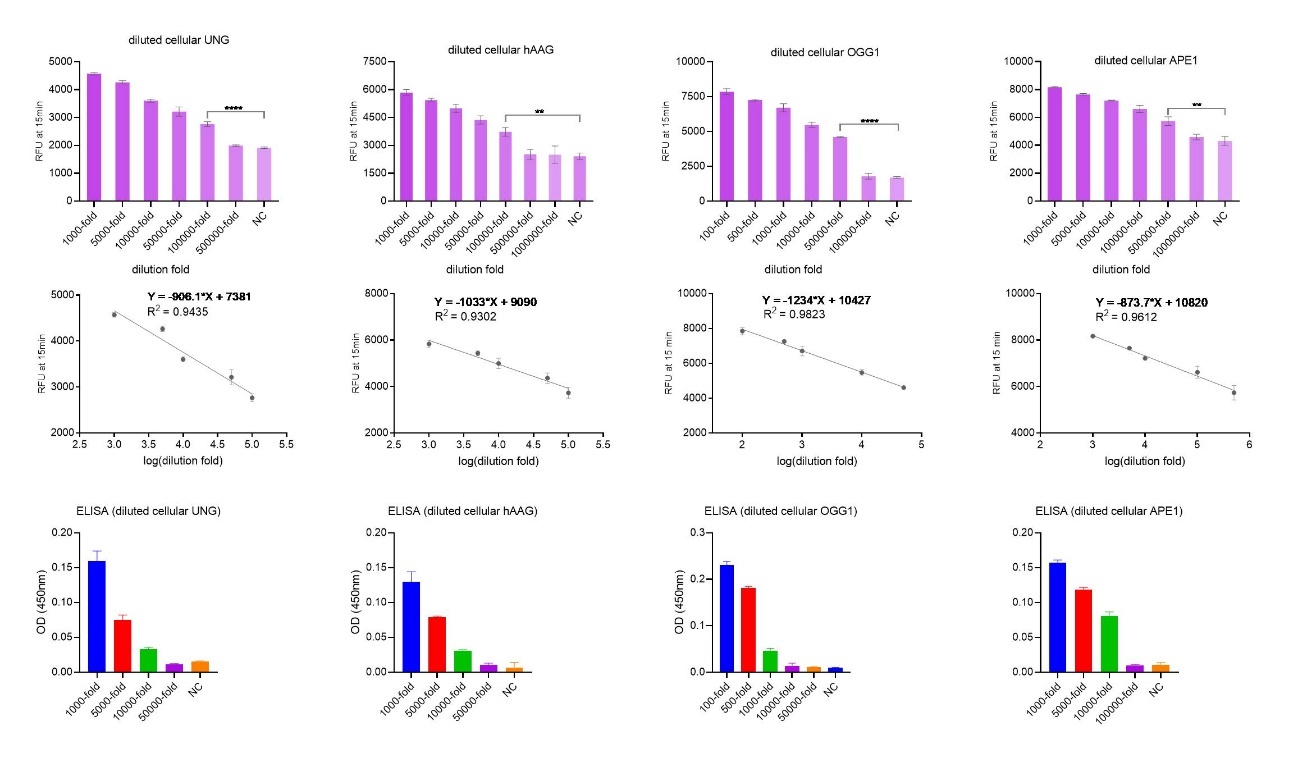


**Figure S14.** The endpoint fluorescence intensity of multiplex BER-Ago assay when adding diluted A549 cellular extracts; The linear relationship between the endpoint fluorescent intensity and the logarithm of dilution fold; the ELISA assay for the detection of diluted A549 cellular extracts.


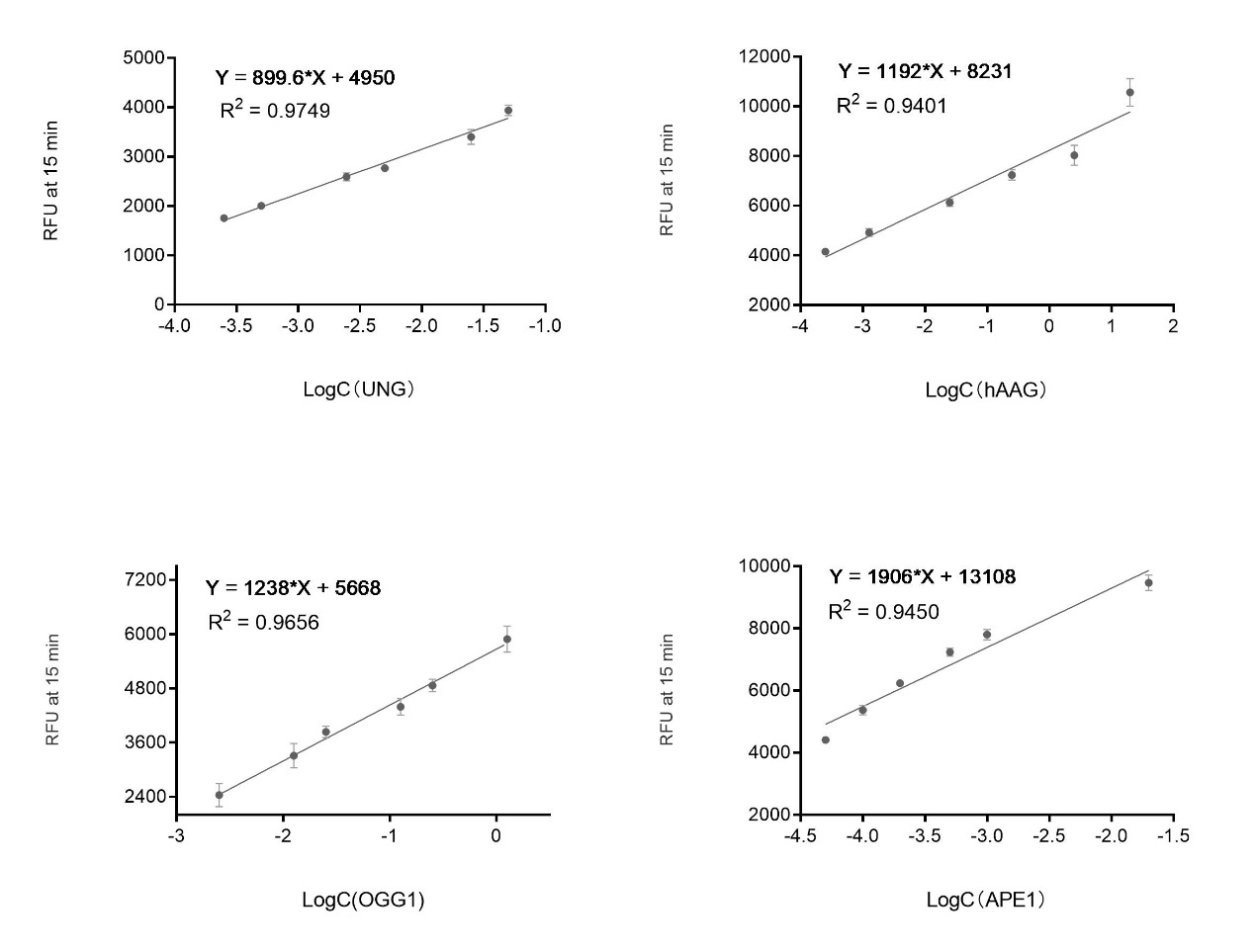


**Figure S15.** The linear relationship between the endpoint fluorescent intensity and the logarithm of BER protein concentration spiked into 10% serum.


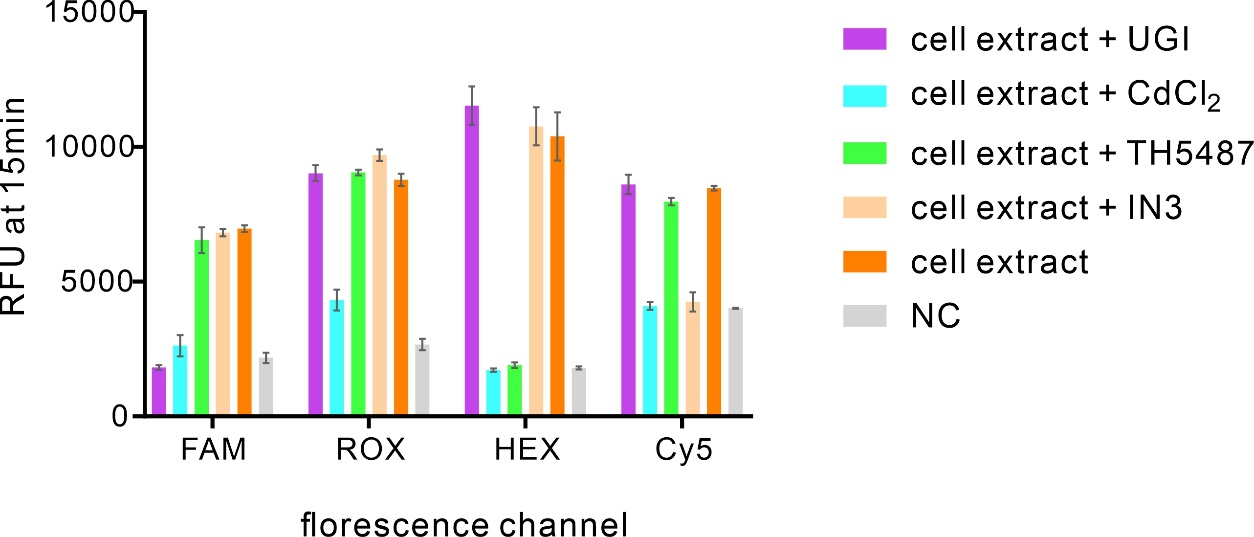


**Figure S16.** Inhibition of cellular BER proteins with different inhibitors. The concentrations of UGI, CdCl2, TH5487 and IN3 were 0.5 U/uL, 150 uM, 0.5 uM and 100 uM, respectively.

1. Hu, J.; Liu, W.; Wang, J.; Qiu, J.-G.; Zhang, C.-y., Simple Mix-and-Read Assay with Multiple Cyclic Enzymatic Repairing Amplification for Rapid and Sensitive Detection of DNA Glycosylase. *Analytical Chemistry* **2021,** *93* (18), 6913-6918.

2. Du, W.; Li, J.; Xiao, F.; Yu, R.; Jiang, J., A label-free and highly sensitive strategy for uracil-DNA glycosylase activity detection based on stem-loop primer-mediated exponential amplification (SPEA). *Analytica Chimica Acta* **2017,** *991*, 127-132.

3. Fan, L.; Liu, W.; Yang, B.; Zhang, Y.; Liu, X.; Wu, X.; Ning, B.; Peng, Y.; Bai, J.; Guo, L., A highly sensitive method for simultaneous detection of hAAG and UDG activity based on multifunctional dsDNA probes mediated exponential rolling circle amplification. *Talanta* **2021,** *232*, 122429.

4. Chen, X.; Cao, G.; Zhang, J.; Deng, Y.; Luo, X.; Yang, M.; Huo, D.; Hou, C., An ultrasensitive and point-of-care strategy for enzymes activity detection based on enzyme extends activators to unlock the ssDNase activity of CRISPR/Cas12a (EdU-CRISPR/Cas12a). *Sensors and Actuators B: Chemical* **2021,** *333*, 129553.

5. Zhang, Q.; Zhao, S.; Tian, X.; Qiu, J.-G.; Zhang, C.-y., Development of a CRISPR-Cas-Based Biosensor for Rapid and Sensitive Detection of 8-Oxoguanine DNA Glycosylase. *Analytical Chemistry* **2022,** *94* (4), 2119-2125.

6. Ding, S.; Yuan, Y.; Dong, J.; Du, F.; Cui, X.; Shi, Z.; Tang, Z., Leveraging CRISPR/Cas12 signal amplifier to sensitive detection of apurinic/apyrimidinic endonuclease 1 and high-throughput inhibitor screening. *Analytica Chimica Acta* **2024,** *1291*, 342212.

7. Tian, G.; Li, W.; Liu, B.; Xiao, M.; Xia, Q., An enzyme-free electrochemical biosensor based on NiCoP@PtCu nanozyme and multi-MNAzyme junctions for ultrasensitive Uracil-DNA glycosylase detection. *Sensors and Actuators B: Chemical* **2023,** *379*, 133224.
